# High-Efficiency Interdigitated Electrode-Based Droplet Merger for Enabling Error-Free Droplet Microfluidic Systems

**DOI:** 10.1021/acs.analchem.4c02376

**Published:** 2024-08-15

**Authors:** Jeong Jae Han, Han Zhang, Yuwen Li, Can Huang, Adrian R. Guzman, Arum Han

**Affiliations:** † Department of Multidisciplinary Engineering, Texas A&M University, College Station, Texas 77843, United States; ‡ Department of Electrical and Computer Engineering, Texas A&M University, College Station, Texas 77843, United States; § Department of Biomedical Engineering, Texas A&M University, College Station, Texas 77843, United States; ∥ Department of Chemical Engineering, Texas A&M University, College Station, Texas 77843, United States

## Abstract

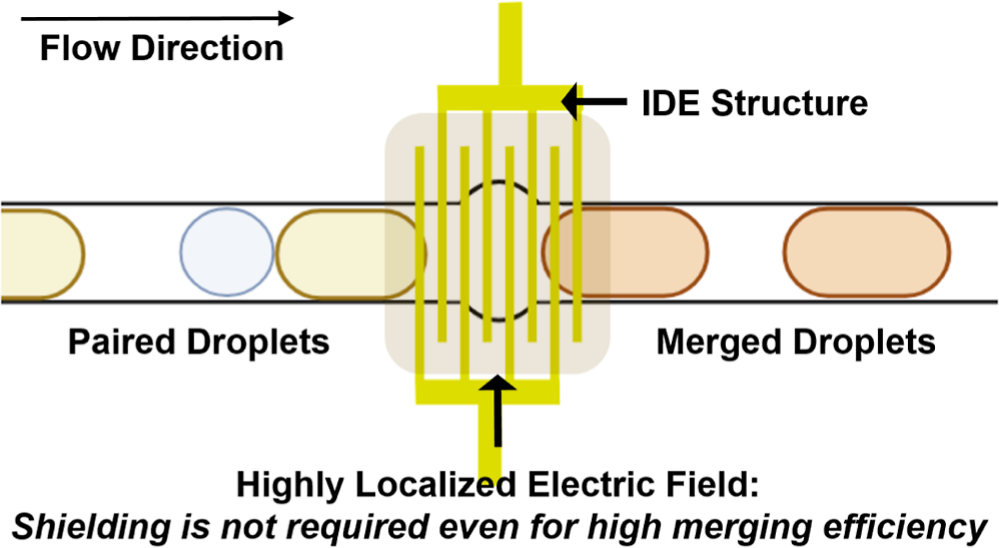

Merging two droplets into a droplet to add and mix two contents is one of the common droplet microfluidic functions with droplet generation and sorting, performing broad ranges of biological and chemical assays in droplets. However, traditional droplet-merging techniques often encounter unsynchronized droplets, causing overmerging or mis-merging, and unwanted merging outside of the desired zone. This is more severe when the incoming droplets to be merged are polydisperse in their sizes, often observed in assays that require long-term incubation, elevated-temperature, and/or multiple droplet processing steps. Here, we developed an interdigitated electrode (IDE)-based droplet merger consisting of a droplet autosynchronizing channel and a merging channel. The autosynchronizing channel provides >95% merging efficiency even when 20% polydispersity in the droplet size exists. The highly localized and enhanced dielectrophoretic force generated by the IDEs on the channel bottom allows droplet merging at an extremely low voltage (4.5 V) and only locally at the IDE region. A systematic evaluation of how various design and operation parameters of the IDE merger, such as IDE finger dimensions, dielectric coating layer thickness, droplet size, and droplet flow speed impact the performance was conducted. The optimized device showed consistent performance even when operating for up to 100 h consecutively at high throughput (100 droplets/s). The presented technology has been integrated into a droplet microfluidics workflow to test the lytic activities of bacteriophage on bacterial host cells with 100% merging efficiency. We expect this function to be integrated into droplet microfluidic systems performing broad ranges of high-throughput chemical and biological assays.

## Introduction

Droplet microfluidic systems have demonstrated great promise in their ability to conduct high-throughput screening assays for a broad range of life science and biotechnology applications, including single-cell studies,^1,2^ sequencing,^3,4^ drug screening,^5−8^ and phage discovery.^9,10^ Highly efficient droplet manipulation serves as a crucial aspect in any droplet microfluidic system, where the efficiency and error in operation often determine the success or effectiveness of a given high-throughput assay. Thanks to the extensive technological advancements made over the past decade in this field, individual droplets can now be efficiently transported,^11,12^ merged,^13,14^ and sorted^15−17^ at very high throughputs with minimal error. Among these fundamental droplet manipulation techniques, controlled merging of droplets at a one-to-one ratio is the basis for adding/mixing reagents and initiating reactions/interactions between different contents in the paired droplets. Droplet merging techniques can be categorized into two domains: passive or active merging methods. Passive merging methods utilize various microstructures, such as micropillar arrays,^18^ widening-narrowing channels,^19^ and meandering channels,^20^ to create a collision–relaxation force between the paired droplets. However, they are unreliable in the presence of surfactants that are commonly used to increase the stability of the droplets. Therefore, active merging methods have become the most commonly utilized methods, since they work efficiently even in the presence of surfactants. Among various active merging methods,^13,14,21^ electric field-based droplet merging provides a precise and high-throughput method for one-to-one droplet fusion with high efficiency, and thus is currently one of the most widely adopted droplet-merging methods.

Among the electric-field-based droplet-merging methodologies,^13^ three-dimensional (3D) electrodes (saltwater and liquid metal)-based^22^ and planar-electrodes-based^23−25^ droplet merging are the most common methods. Despite the high performance of these methods, they still have several challenges, including relatively high operational voltage requirements or low droplet merging throughput/efficiency. 3D electrodes-based droplet merging typically requires a moderate to high voltage (200–1000 V) due to a large electrode-to-electrode gap inherent to this approach.^13^ Generally, to achieve higher throughputs, higher operational voltages are required to merge droplets. In this case, even in the presence of shielding electrodes that prevent the high electric field from affecting other areas of the microfluidic system, it is unavoidable that the electric field affects a much broader area. As a result of this, merging could occur at random places in the microfluidic channels whenever two droplets come too close to each other. Planar-electrode-based droplet-merging method can achieve droplet merging at significantly low voltages (5–20 V); however, the throughput is relatively limited (<100 droplets/s) because of the relatively weak electric field strength generated by a pair of planar electrodes, and efficiency remains relatively low (<90%).^13,23^

Here, we describe the development of a droplet merging device that uses interdigitated electrode (IDE) arrays to overcome the aforementioned challenges. In the IDE array design, because the electrode is a surface electrode, the generated electric field is naturally highly confined to the surface, and also because the electric field is generated between two opposing electrodes that are close together (typically less than a few micrometers distances), they are highly confined between the electrode fingers. This enables a highly localized electric field to be generated, meaning that droplet merging occurs only when the droplets are on top of the IDE array but not outside of the IDE array region. Because the electric field needs to be only generated between the IDE fingers (typically a few micrometers gap), only a few volts are needed, significantly lower than the voltages needed in conventional droplet merging devices. Thus, droplets outside of the merging region will not experience any stray electric field, meaning that no additional electric field shielding or grounding is required to prevent unwanted merging in other areas of the device, where such shielding remains imperfect anyway and where it also limits the design of other droplet manipulation components in the overall droplet microfluidic system. This merging design was integrated with a recently developed droplet autopairing scheme^14^ followed by a repeated expansion-narrowing microchannel design that further improves the efficiency of one-to-one droplet pairing and synchronization especially when the droplet sizes are polydisperse. The developed design was tested for its droplet merging efficiency in direct comparison to that of a conventional 3D electrode-based droplet merger. Design and operational parameters such as IDE finger distance, dielectric coating layer thickness, volumetric ratio of paired droplets, throughput, presence of the repeated expansion-narrowing channel and cleaving junction, and longevity of successful merging efficiency were tested between the developed IDE droplet merger and the conventional 3D electrode-based droplet merger. Finally, the device was tested for a real biological assay, which involved assessing the lytic activities of bacteriophages on bacterial host cells.

## Materials and Methods

### Device Fabrication

The device comprises an IDE structure on a borosilicate glass substrate, with a microfluidic channel placed on top of it. The detailed fabrication procedure is illustrated in the Supporting Information, Figure S1. In brief, the IDE structures were created by using metal evaporation, followed by photolithography and metal etching. The microfluidic device was fabricated in polydimethylsiloxane using conventional soft lithography technology.^26^ These two layers were then bonded together after oxygen plasma treatment to facilitate strong bonding.

### Experimental Setup for Evaluating the Droplet-Merging Performances

To fully characterize the droplet merger device, 45 μm diameter droplets were generated by a T-junction droplet generator using fluorinated oil (Novec 7500, 3M) containing 2% (w/w) surfactant (Pico-Surf, Sphere Fluidics, Cambridge, UK) and black color dyed DI water. To achieve a throughput of 50 merged droplets/s, flow rates of the target cell stream, reflow droplet stream, and spacing oil stream were set to 100, 45, and 350 μL/h, respectively. The electric field was induced by applying a sinusoidal electric signal having a frequency of 6.5 kHz using a function generator (DG4202, RIGOL, Portland, OR) and amplifying the signal using a high-voltage amplifier (model 2210, TREK INC, Lockport, NY). The “operational voltage” is defined as the minimum required voltage that can achieve >95% droplet-merging efficiency, which was set as the minimum efficiency for successful droplet merging.

The effect of the repeated expansion-narrowing channel on the droplet-pairing efficiency was investigated by comparing two identical devices with and without the repeated expansion-narrowing channel, where different throughputs from 5 to 50 droplets/s were tested at voltages between 0 and 20 V.

Finally, the size distribution of the merged droplet was analyzed (10 μm finger distance and 500 nm of Si_3_N_4_). The throughput of the droplet was 50 droplets/s, droplet-merging events were recorded by a high-speed camera (C14440-20UP, Hamamatsu Photonics, Shizuoka, Japan), and the resulting merged droplet sizes were calculated by MATLAB (Mathworks, Natick, MA). The detailed methodology using MATLAB for merged droplet size calculations is included in the Supporting Information file.

### Cell and Reagent Preparation for the In-Droplet Bacteriophage-Based Bacterial Host-Killing Assay

Bacteriophage T4, which has lytic activities against *Escherichia coli* MG1655 (ATCC 700926) as the host cell,^27,28^ was selected as the model phage. *E. coli* cells were cultured in Luria–Bertani (Sigma-Aldrich) culture medium at 30 °C in a shaking incubator (150 rpm) after inoculating cells from a frozen stock. After an OD_600_ of 0.2 was reached, cells were stained using 1× green fluorescence dye (BactoView, Biotium, CA, USA) to observe the growth of cells in each droplet. After that, droplets were generated with approximately 10–20 *E. coli* cells per droplet (45 μm diameter). After the generation, the droplets were directly reflowed into the developed IDE merger device as well as into a conventional merger device that includes the droplet cleaving junction for direct comparison. Here, T4 phage at a concentration of 1 × 10^10^/mL was flown into the system. To ensure complete and fast killing of the target bacterial cells by T4 phage, the final multiplicity of infection of T4 phage against *E. coli* was set to 10. After the droplets containing *E. coli* and T4 phage were merged, the merged droplets were incubated at 30 °C for 12 h. The merged and cultured droplets were then reflown into a droplet imaging chamber^29,30^ to evaluate the performance of the droplet merging device. The expectation is that the growth of *E. coli* will be inhibited in the presence of T4 phage and thus low green fluorescence will be observed since the number of *E. coli* cells will remain constant, while droplets containing only *E. coli* cells will show an increased number of cells due to growth and thus lead to a high green fluorescent intensity. The error rate was calculated as the number of small, highly fluorescent green droplets divided by the total number of droplets in the imaging chamber, since this indicates instances where an *E. coli*-containing droplet did not successfully merge with a T4-containing droplet, resulting in no lytic activity.

## Results and Discussions

### Device Design and Working Principle

**Figure 1:**
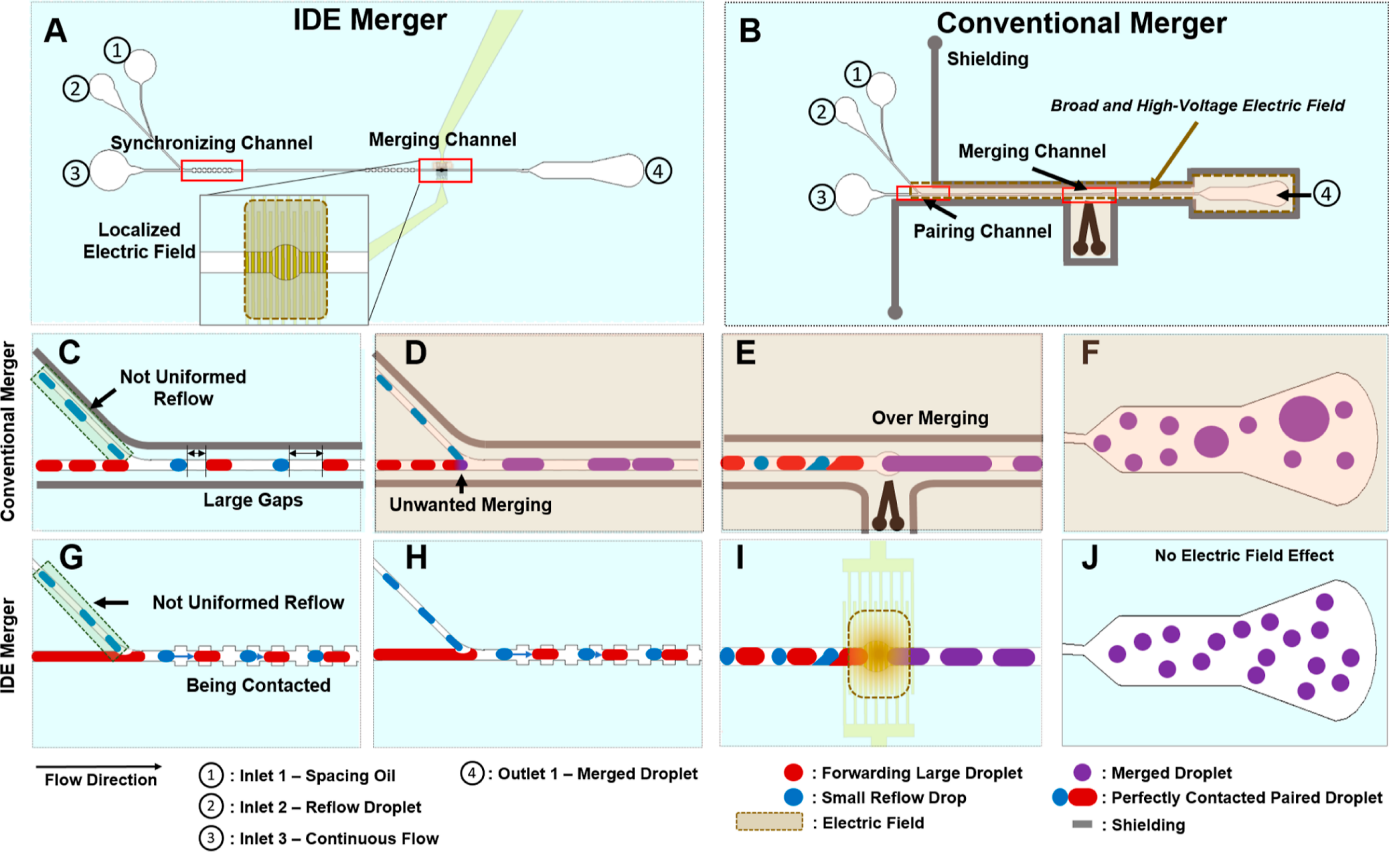
Design of the IDE droplet merger device in comparison to a conventional droplet merger. (A) Schematic of the IDE merger device. (B) Schematic of a conventional merger device. The gold electrodes around the microchannels indicate shielding electrodes to prevent unwanted droplets from merging outside of the merging zone. (C–F) Various failure modes that are commonly seen in conventional droplet mergers. (C) Droplet-to-droplet gap not consistent, causing the two droplets desired to be merged to be too far apart, resulting in failed droplet merging. (D) Unwanted droplet merging at the 45° junction and outside of the merging zone even when a shielding electrode is used due to the relatively high voltage required for droplet merging. (E,F) Overmerging of more than two droplets due to the electric field not sufficiently localized: (E) is in the merging region; (F) is in the droplet outlet region. (G–J) Illustration showing how the developed IDE merger overcomes each of the failure modes of a conventional merger device. (G) Autosynchronization of droplet pairs to minimize droplet-to-droplet distances. (H) No merging at the 45° junction. (I,J) No overmerging at the (I) merging channel and the (J) outlet.

The working principle of the developed IDE-based droplet merger device and how the design overcomes the challenges of a conventional droplet merging are illustrated in Figure 1 and S2. The IDE-merging device (Figure 1A), which measures 11 mm in width and 26 mm in length, contains two main droplet manipulation channels: a droplet synchronizing channel and a droplet merging channel. The synchronizing channel consists of (**1**) as a droplet-cleaving junction and (**2**) as a repeated expansion-narrowing channel section. At the cleaving junction, the reflow of the droplet stream cleaves the continuous aqueous flow to form an autopaired one-to-one droplet stream, as previously reported.^13^ The repeated expansion (130 μm width)-narrowing (70 μm width) channel section as part of the synchronizing channel will further minimize droplet-to-droplet distances and allow contact between the paired droplets to maximize the efficiency of droplet merging. The merging channel has an array of IDE fingers such that multiple induced overlapping electric field collectively create an enhanced field effect to merge each pair of droplets (Supporting Information, Figure S3). In addition, the channel in this merging region widens to allow the two autosynchronized droplets to experience a collision force. Since droplet merging primarily depends on two forces in the merging channel, namely collision–relaxation force between droplets^31^ and DEP force,^13^ this design provides synergy of providing both forces in the same location, lowering the energy barrier needed for successful droplet merging and maximizing the efficiency (Supporting Information, Movie S1), as well as achieving merging even when a small gap between the two droplets desired to be merged exists (Supporting Information, Movie S2).

As can be seen in Figure 1, reflow droplets are introduced through inlet 2, separated by a spacer oil flow from inlet 1, and subsequently directed into the continuous flow phase at a 45° angle. This scheme allows the reflow droplets to cleave the aqueous stream (inlet 3), where synchronized droplet pairs emerge upon reaching the expansion channel. The device has a channel height of 50 μm, a spacing oil channel width of 30 μm, a droplet reflow channel width of 45 μm, and a main channel width of 70 μm. The IDE finger width and finger-to-finger distance are 10 and 10 μm, respectively.

**Figure 2:**
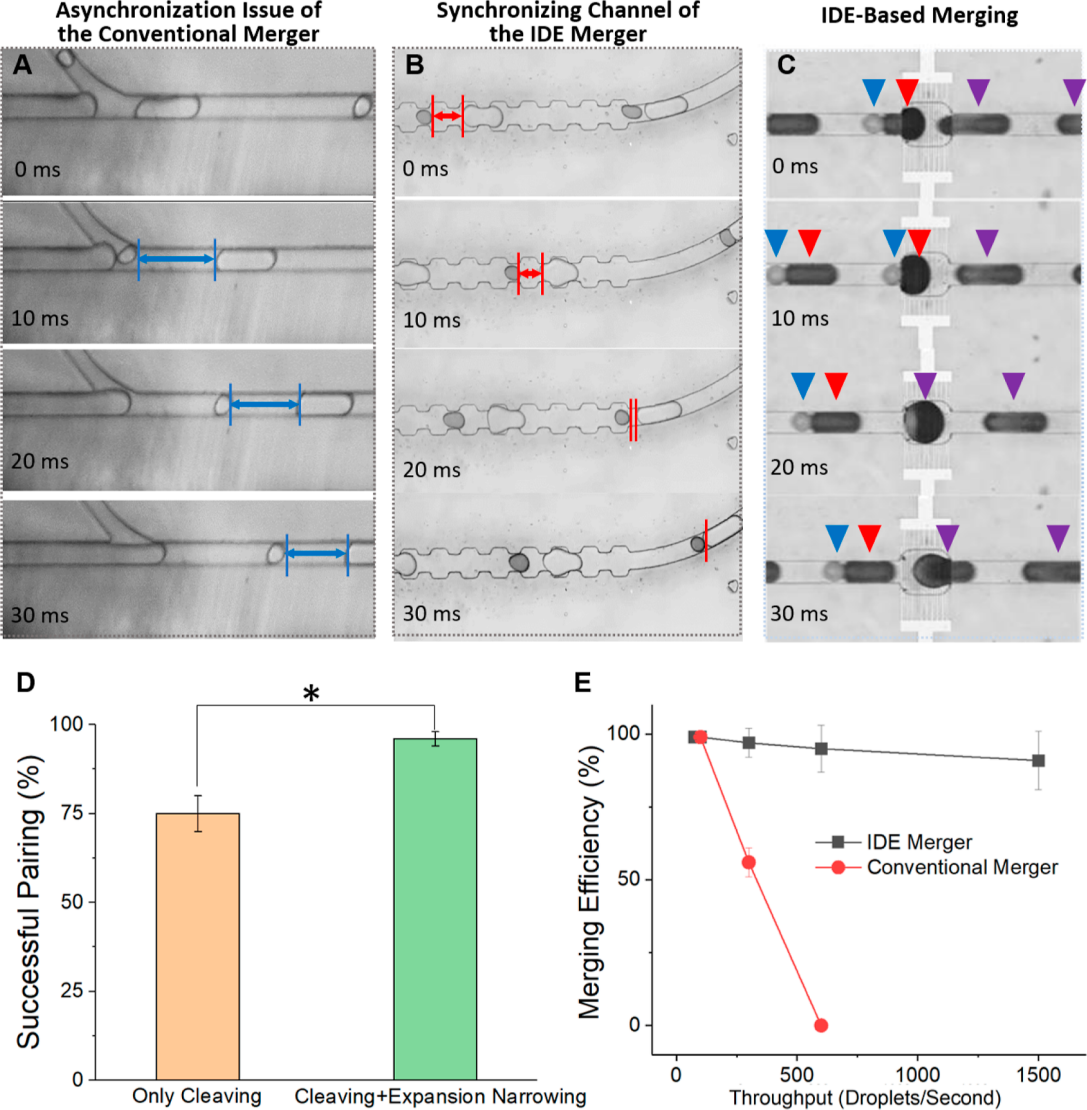
Performance of the IDE merger in enabling close droplet-to-droplet distances of paired droplets followed by successful droplet merging, compared to previously published conventional droplet merger device. (A–C) Frame-by-frame images of (A) the desynchronization issue of the conventional merger, where a gap remains between paired droplets before they enter the merging channel, (B) the synchronizing channel of the IDE merger where the gap was successfully reduced before they were entering the merging channel, and (C) IDE-based merging. (D) Comparison of successful pairing ratios with and without the expansion-narrowing channel (*n* = 300 and *p* < 0.01). (E) Merging efficiencies of the IDE and conventional droplet merger at various throughputs.

The schematic of a conventional merger is illustrated in Figure 1B. The conventional merger, with structural similarity to the developed IDE merger for direct comparison, comprises of a pairing channel and a merging channel, along with liquid metal-based 3D electrodes for droplet merging and electric field shielding as is commonly done.^14^ Due to the similar structural characteristics of the two designs, all microfluidic flow conditions were kept identical, with the only distinction being that droplets reflow from inlet 3 instead of a continuous flow. Several failure modes of this conventional merger device are illustrated in Figure 1D–F. First, in any given droplet library maintaining strict droplet size, monodispersity is quite challenging, especially after multiple droplet manipulation steps such as incubation, reflow, and transition. Thus, in most cases, there will be a small percentage of paired droplets that have considerably larger gaps between them due to the polydispersity of reflowed droplets (Figure 1C). Second, a high operational voltage is generally required to achieve high-throughput merging. However, this leads to a stray electric field outside of the merging zone even in the presence of shielding electrodes, covering a much larger area of the fluidic channel outside of the desired merging zone with a relatively high electric field (orange color area in Figure 1D–F). This results in two commonly observed failure modes: (1) merging occurring at unwanted locations (Figure 1D,F) and (2) overmerging of more than two droplets (Figure 1E). For example, unwanted merging at the pairing channel can be observed when the blue reflow droplets meet the red reflow droplets at the junction under the influence of a stray electric field (Supporting Information, Movie S3). In the second example, merged droplets can be merged again with another droplet even if there is some spacing between them since the shape of the droplets becomes distorted due to the strong electrical field and high shear stress (streaming effect).^29^

To solve the aforementioned challenges, the IDE merger incorporates a repeated expansion-narrowing channel section after the droplet cleaving section, where such a channel geometry reduces the droplet-to-droplet distance of a paired droplet when there is a gap between the two droplets to be merged (Figure 1G). The repeated expansion–narrowing channel section allows the spacing oil to bypass around the larger front droplet at the wider point of the channel while the rear smaller droplet is squeezed by the preceding narrowing segment. This phenomenon reduces the amount of oil between droplets and minimizes the distance between the paired droplets. Since the IDE-based droplet merger device requires a significantly lower operational voltage (40 times lower^14^), merging at unwanted locations and overmerging are not observed (Figure 1H–J). An added benefit is that since the electric field is confined and the operational voltage is much lower, no shielding electrodes are needed, significantly simplifying the overall device design.

### High-Efficiency Droplet Synchronization and IDE-Based Droplet Merging

High merging performance relies heavily on the monodispersity of input droplets, which is difficult to realize in realistic droplet operation scenarios. A gap between paired droplets can be created due to reflowing polydisperse droplets reflowing. Figure 2A shows the synchronization section of our previously published conventional droplet cleaving-based merger device,^13^ which has a straight channel and where significant droplet-to-droplet distances in paired droplets exist when the input droplets are polydisperse. In contrast, in the developed design having the repeated expansion–narrowing channel, the initial paired droplets with gaps could be minimized so that the paired droplets are right next to each other by the time they exit this expansion-narrowing channel (Supporting Information, Movie S4). Following this section, the channel widens, where the IDE merging section is located (Figure 2C). Here, the well-synchronized droplets (from Figure 2B) were merged efficiently under the effect of DEP force and collision-relaxation force (Supporting Information, Movie S5). Figure 2D compares the droplet pairing rate with and without the expansion–narrowing section using cell culture medium (Luria Broth)-encapsulated droplets. The droplets were then incubated overnight to mimic an in-droplet cell cultivation step, where this converted the initially highly monodisperse droplets (droplet diameter: 71.6 ± 7.2 μm) into polydisperse droplets (80% of the droplets intact, 20% of the droplets larger or smaller, droplet diameter: 73.2 ± 14.2 μm), since merging or evaporation of droplets occurred during this incubation steps. The size distribution of pre- and postincubation droplets are shown in Supporting Information, Figure S4. The result shows that the final droplet pairing ratio increased by approximately 20% with the expansion–narrowing microchannel structure. Since the characteristics of the aqueous contents in the droplets, such as viscosity or pH level, do not significantly affect the droplet behaviors, different types of culture medium can also be utilized without affecting the merging efficiency.

The performance of the IDE merger at different throughputs was also compared to a conventional droplet merger device^13^ (Figure 2E). The design of the conventional droplet merger device used here is shown in the Supporting Information, Figure S5. As can be seen in Figure 2E, at a relatively low throughput (<100 droplets/s), the merging efficiency of both methods were comparable. However, at higher throughputs (>300 droplets/s), the efficiency of the conventional droplet merger device significantly dropped to around 50% at 300 droplets/s, and then further lower to around 20% at a throughput of 600 droplets/s. This drop was primarily caused by unwanted droplet merging or overmerging under high operational voltage (sinusoidal wave signal, >240 V @ 6.5 kHz after amplification) required at higher throughput. Unwanted droplet merging could also occur where the strong electric field covers droplets having smaller gaps, hence higher spacing oil flow rate is needed to increase the gaps between the paired droplets to avoid unwanted merging. However, as the Supporting Information, Figure S6A illustrates, the IDE droplet merger device can perform under extremely small droplet pair-to-pair distances due to its highly localized electric field used for droplet merging. On the other hand, a conventional droplet merger device having 3D electrodes requires a larger droplet pair-to-pair distance to avoid overmerging due to its strong and broad electric field (Supporting Information, Figure S6B). The overall operation of the device is shown in the Supporting Information, Movie S6.

**Figure 3:**
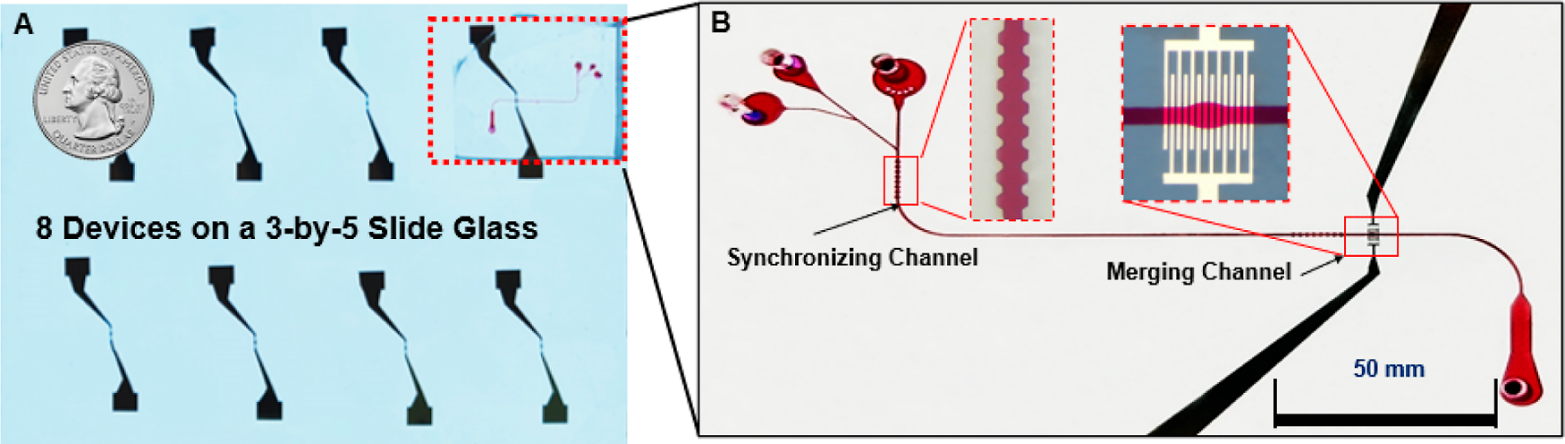
Images of an eight-device array assembled and ready for use. (A) Eight IDE patterns on a 3 × 5 in. borosilicate glass substrate. (B) A single device diced from the glass substrate shown in (A).

Another advantage of the IDE-based droplet merger device is simplification and reduction of the fabrication steps and cost, such that precise quality control and easier mass fabrication are possible. The standardized fabrication steps of the IDE droplet merger device, which does not require the 3D shielding electrodes, provide more precise quality control and easier fabrication since liquid metal injection is the only part that is difficult to control consistently due to its manual fabrication step.^13^ The lack of a shielding electrode requirement also makes the overall device footprint much smaller (Figure 3A/B). Furthermore, to further simplify the fabrication procedure and lower the material cost, indium tin oxide^32^ can be used as substitute methods^33−35^ instead of Ti/Au electrodes.

### Characterization of Droplet Merging Efficiency and Operating Voltage

**Figure 4:**
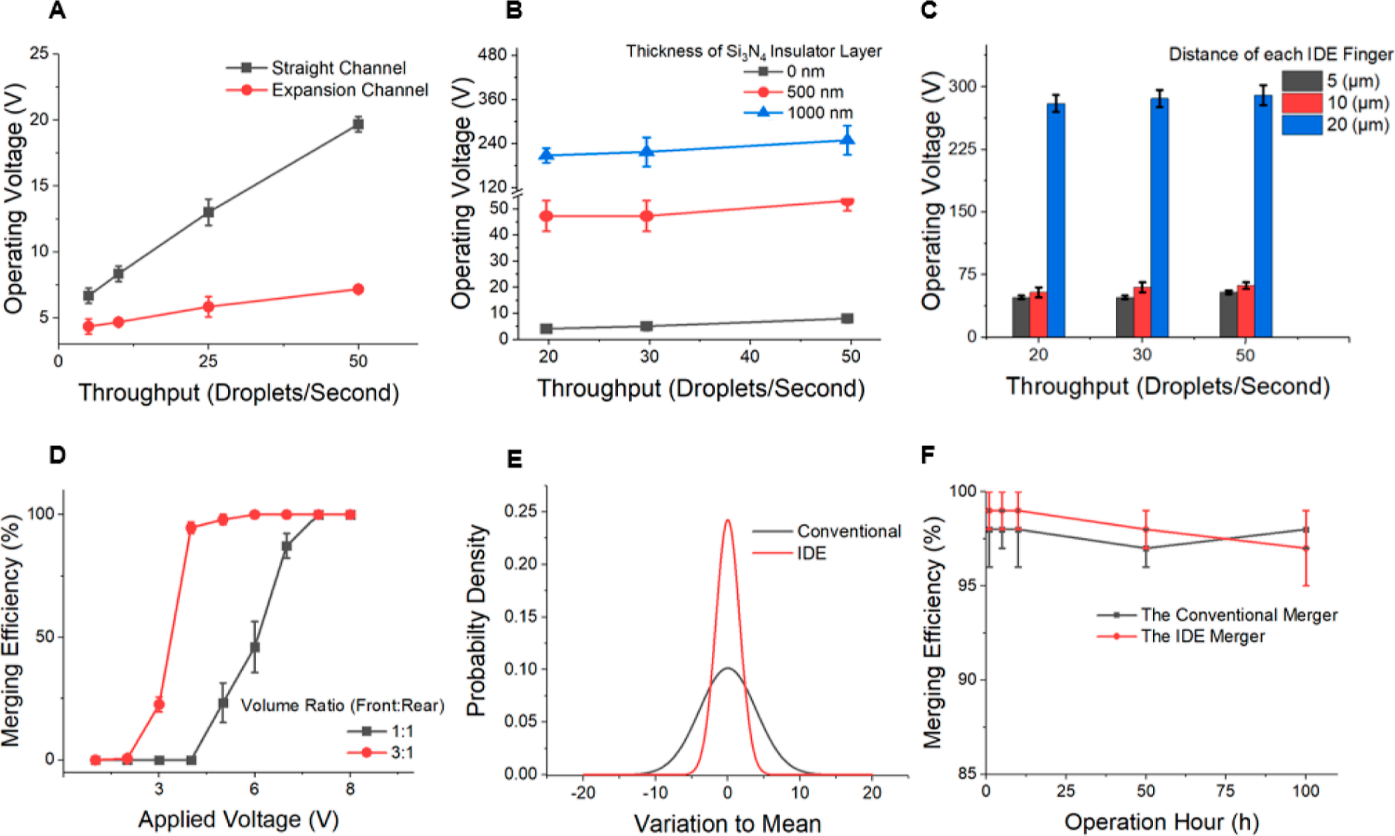
Characterization of the device performance. (A–C) Required operating voltages, which are defined as the minimum required voltage to achieve 95% merging efficiency for (A) merging in straight and expansion channels at various throughputs, (B) different thicknesses of the Si_3_N_4_ insulation layer (0, 500, and 1000 nm), and (C) various throughputs for IDE finger distances of 5, 10, and 20 μm. (D) Correlation of voltage vs efficiency for droplet pairs with 1:1 and 3:1 volumetric ratio (front vs rear). (E) PDF showing the size distribution of the merged droplets. (F) Merging efficiencies of the IDE and conventional mergers over long term (100 h) device operation.

The impact of the expansion channel on the IDE pattern, thickness of the insulator layer, properties of the IDE pattern, volumetric ratio of paired droplets, and operation hours on the performance of the IDE-based droplet merger were characterized. First, the minimum operation voltages needed for droplet merging with and without the expansion channel in the droplet merging region under four different throughputs were investigated. Here, a volumetric ratio of 3:1 between the front and rear droplets was used. In the device with the expansion channel, the required operational voltage was 4 V at five droplets/s, which is much lower than the 7 V required for the straight channel design (Figure 4A). The result indicates that the collision-relaxation force created by the droplets suddenly slowing down due to the channel being expanded could significantly reduce (75% lower) the required operational voltage. The differences in required voltage between the straight channel and the expansion channel were further increased as the throughput (droplet/s) increased. For example, at 50 droplets/s, the IDE-based droplet merger with the expansion channel could be operated at a relatively low voltage of 7 V, compared to 20 V needed without the expansion channel. Importantly, this is 129 times lower than the published results of 3D liquid metal or 2D planar electrode-based droplet merger devices.^14^

Although merging at very low voltages has been achieved, the IDEs can be worn out relatively quickly (within hours) since droplets come into direct physical contact with the IDEs. The Supporting Information, Figure S7, shows a worn IDE after 4 h of continuous droplet merging operation. To solve this issue, a Si_3_N_4_ insulator layer was employed as a protective layer on the electrodes. However, as this coating layer increases the dielectric constant, at the same applied voltage the generated electric field becomes much weaker, and thus needs to be compensated for. Figure 4B shows the correlation between the Si_3_N_4_ coating thickness and the required operational voltage. For example, the operational voltage of 48 V required for a thin layer (500 nm) is 4.5 times lower than the 214 V needed for the thicker layer (1000 nm), in contrast to the 4 V needed when no insulating layer is used. Note that this relationship is not linearly correlated due to the strength of the electrical field being attenuated exponentially as the layer thickness increases.^36,37^

The strength of the electrical field can be affected by not only the thickness of the Si_3_N_4_ insulator layer but also the finger-to-finger distance of the IDE structure. Figure 4C shows the effect of the center-to-center finger distance of the IDEs on the operation voltage. As the distances between fingers increased, a higher voltage was required to successfully merge droplets. However, the difference between 5 and 10 μm finger distance was minimal (48 and 54 V, respectively), indicating that 10 μm finger distance would be sufficient for operation (in contrast to 20 μm distance requiring 280 V). The minimum voltage requirement was tested at three different throughputs of 20, 30, and 50 droplets/s. The tendencies of required voltage for different finger distances were the same where different droplet throughputs were employed and where the required voltages were higher as droplet throughputs increased. The larger gaps between each finger cause a larger radius of the DEP fields and lower the strength of the field such that the force to attract the paired droplets to be merged is degraded. Also, the strength of the DEP field is negatively related to the square of finger distance,^32^ and attenuation from the origin of the DEP field to the droplet degraded the strength of the DEP field such that the required voltage was not linearly related. Regardless, the minimum required voltage even at a higher throughput did not change significantly (48 vs 54 V).

The required voltages when paired droplets having different volumetric ratios of 1:1 and 3:1 were also investigated. As the experimental results show (Figure 4D), the 1:1 volume ratio of front and rear droplets required higher voltage (5 V) to achieve merging, compared to the 3 V required for the 3:1 volumetric ratio (front and rear droplets). The higher voltage requirement of 1:1 volumetric ratio of droplets can be caused by two major reasons: (1) the larger size difference between front and rear droplets leads to more speed difference and hence introduces a larger collision force; (2) the surface tension of larger droplets is lower and hence the merging energy threshold is lower relative to smaller droplets.

All droplet-manipulating functions in general are designed to work under a certain range of droplet sizes to perform optimally, and thus, a large size variation in droplet size can affect the efficiency of droplet manipulation. Therefore, it is necessary to validate the stability of the developed droplet merging function at various droplet sizes. Figure 4E shows the probability density function (PDF) of the size of the merged droplets resulting from the IDE merger device, in comparison to that of the conventional merger device. The standard deviation calculated from the merged droplets from the IDE merger device and the conventional merger device were 1.6 and 3.9 μm, respectively (relative standard error 3.8 vs 7.6%). This result demonstrates that the proposed system could achieve a relatively uniform merged droplet size.

Finally, to assess the robustness of the design, the IDE merger was tested under continuous operation for up to 100 h (Figure 4F) at a throughput of 100 droplets/s, and its performance was compared with that of the conventional merger. Merging efficiencies of both designs were 98–99% on day 1 and did not show any fluctuation until the end of the experiment (98–99%). It is noteworthy that both designs demonstrated sufficient robustness for conducting extensive biological screening assays. Additionally, electrodes coated with a 500 nm thick Si_3_N_4_ layer are deemed suitable for long-term and high-throughput processing. The only scenario that could affect the performance and reliability of the device is the degradation of the IDE structure due to an excessively high operational voltage caused by user error. However, aside from this user error, the developed IDE droplet merger is robust and can maintain its performance and reliability for long-term operation.

### Application to Bacteriophage-Based Targeted Lysis of Bacterial Host Cells in Droplets

**Figure 5:**
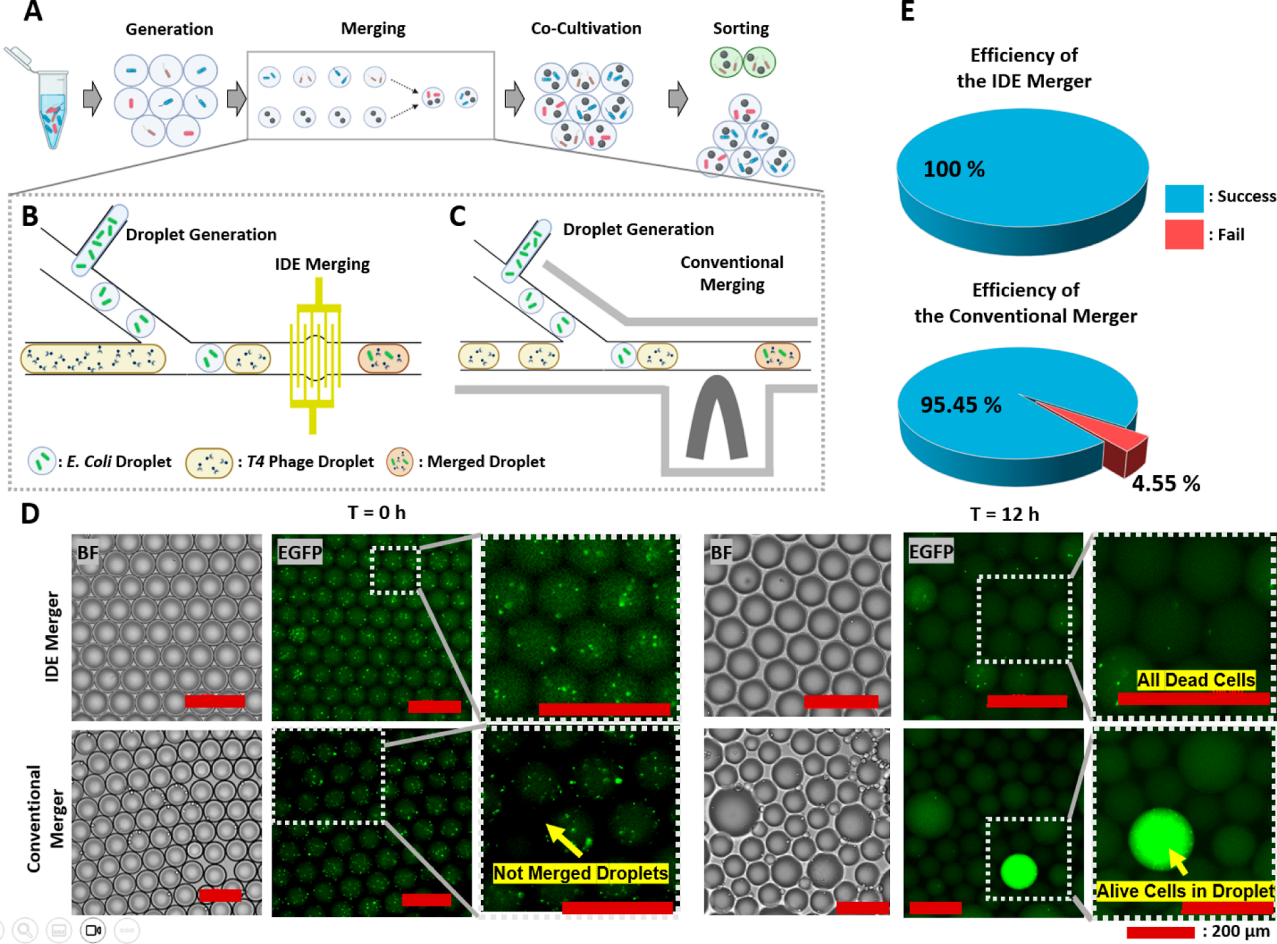
Mock bacteriophage screening against target bacterial cells of interest using the developed low-error droplet merging method. (A) Overview of the droplet microfluidics-based workflow of bacteriophage screening. (B,C) Illustration showing workflows of the droplet merger device using the (B) developed IDE merger device and (C) conventional merger device. (D) Bright field (BF) and green fluorescent field microscopic images of the collected droplets after the droplet merging event, before and after 12 h cultivation (scale bar = 200 μm). (E) Statistical analyses of droplets processed through the IDE merger compared to that of the conventional merger (*N* = 500).

Bacteriophages are the most common biological entities in nature and have been shown to effectively fight and destroy multidrug-resistant microbial pathogens.^38^ In contrast to the vast number of phages believed to exist, current phage isolation and screening methods face challenges associated with low throughput and time-consuming processes. Here, we describe a droplet microfluidics workflow (Figure 5A) to conduct analyses of T4 phages, which specifically target and lyse *E. coli* MG1655 cells (ATCC 700926). First, *E. coli*-encapsulated droplets were generated (Figure 5B,C). Second, paired *E. coli* droplets and T4 phage-encapsulated droplets were created, followed by paired droplet merging. Here, the newly developed IDE-based droplet merger device and the conventional droplet merger device were compared to assess the impact of droplet merging efficiencies on the outcome of the biological assay (Figure 5B vs 5C). Finally, the merged droplets were incubated for 12 h before imaging. After coincubation of *E. coli* and T4 phages, successfully merged droplets should exhibit a low fluorescent intensity as bacterial cells are dead or bacterial growth is significantly inhibited. Unsuccessfully merged droplets should exhibit a high fluorescent intensity due to rapid bacterial growth in the absence of T4 phages. As seen in Figure 5D, the smaller (45 μm in diameter) and brighter droplets represent those that failed to merge. In contrast, most of the other droplets had a diameter of around 90 μm, indicating a successful merging event. Comparing the two conditions after 12 h of culture, it can be seen that the droplet size variability is significantly lower in the case of the IDE merger device. An example of an unmerged droplet (droplet much smaller) showing high *E. coli* growth (represented by high green fluorescence intensity) can also be seen (Figure 5D, 12 h conventional merger). Figure 5E shows that the rates of successful merging of the IDE merger and the conventional merger were 100 and 95.45% (22 failure events occurred out of *N* = 500), respectively.

In the case where the rate of hit is relatively low (e.g., 0.1%), droplets that were not merged (4.55%) will result in 455× false positives compared to the true positive (0.1%), causing the sorted droplet population to contain only 0.2% of real “hit” droplets. As a demonstration, in the bacteriophage screening assay (Figure 5), where one million bacterial cells are screened, a platform that runs at 95.45% efficiency will result in >90,000 unmerged/miss-merged droplets, which can severely impact the downstream processes and could significantly reduce the value of such large-scale high-throughput screening processes. Thus, the developed IDE-based droplet merger could indeed substantially improve the efficiency of the droplet microfluidics-based screening assays. Overall, the IDE merger provides a highly efficient and effective method for droplet microfluidics applications at single-cell resolution and high throughput. By merging millions of independent reactions in the form of microfluidic droplets with minimum error, this technique allows simultaneous testing of a multitude of interactions, significantly increasing the throughput compared to traditional methods. Additionally, this presented IDE droplet merger provides reliable merging starting from 4 V of operational voltage, which is extremely low. The electric field generated by the IDE structure does not cause electrical interference with sensitive biological assays, as demonstrated in our previous publication.^26^

## Conclusions

The work herein shows an IDE-based droplet merger consisting of a droplet synchronizing channel and an IDE-based droplet merging channel. Implementation of the repeated expansion-narrowing channel combined with the droplet cleaving scheme improved the successful droplet pairing ratio by ∼20% compared to that of the conventional merger (75 vs 96%) when the reflow droplet input was polydisperse (73.2 ± 14.22 μm). The IDE droplet merger generated a highly localized electric field on the merging channel through the surface IDEs, and the expansion channel on the IDEs reduced operating voltage to as low as 4.5 V (at 10 droplets/s). Due to the highly localized electric field, the IDE droplet merger also required only a small minimum droplet-to-droplet distance in comparison to that of the conventional droplet merger, allowing droplets to achieve higher throughput at the same flow rate. To validate the stability of the developed droplet merging device, the standard deviation of the merged droplets from the IDE droplet merger device and the conventional droplet merger device were compared, showing that the relative standard error of the merged droplet size from the IDE merger was only 3.8%, compared to 7.6% from the conventional merger device. Continuous operation of the device for up to 100 h demonstrated that the performance had no degradation until the end of the experiment (droplet-merging efficiency maintained at ∼98–99% throughout the assay). Finally, the developed system was applied to an in-droplet bacteriophage assay that involved droplet merging between T4-containing droplets and *E. coli*-containing droplets. The result shows that the rate of successful merging of the IDE merger was 100% (compared to 94.5% from the conventional merger), essentially eliminating false positives and false negatives stemming from droplet operation. This new merging method vastly outperforms conventional merging schemes and can be easily integrated into a broad spectrum of droplet microfluidics-based systems, all while simplifying the fabrication steps and cost.

## Supporting Information Available

Device fabrication, microfabrication of the IDE structure, fabrication of the microfluidic device, experimental setup for evaluating the droplet-merging performances, cell and reagent preparation for the in-droplet bacteriophage-based bacterial host-killing assay, calculation of droplet size using a MATLAB script (MP4)
(MP4)
(MP4)
(MP4)
(MP4)
(MP4)
(PDF)
