# Supplementary material for: High-Efficiency Interdigitated Electrode-Based Droplet Merger for Enabling Error-Free Droplet Microfluidic Systems
Source: Anal Chem. 2024 Aug 15;96(34):13906–15. doi: 10.1021/acs.analchem.4c02376 (PMC11359384; doi:10.1021/acs.analchem.4c02376)
Supplement: Supplementary file 7 — ac4c02376_si_007.pdf [file ac4c02376_si_007.pdf]

High-efficiency Interdigitated Electrode-based Droplet Merger for Enabling Error-Free Droplet Microfluidic Systems

Jeong Jae Han[1] †, Han Zhang[2] †, Yuwen Li[2], Can Huang[2], Adrian R. Guzman[2], Arum Han[2,3,4] *

1 Department of Multidisciplinary Engineering, Texas A&M University, College Station, Texas 77843, USA

2 Department of Electrical and Computer Engineering, Texas A&M University, College Station, Texas 77843, USA

3 Department of Biomedical Engineering, Texas A&M University, College Station, TX 77843, USA

4 Department of Chemical Engineering, Texas A&M University, College Station, TX 77843, USA

†These authors contributed equally to this work.

* This author (Arum Han, arum.han@ece.tamu.edu) is the corresponding author.

This PDF file includes:

Supplementary Text

Figures S1 to S7

Legends for movies S1 to S6

Other Supplementary material for this manuscript includes the following:

Movies S1 to S6

SUPPLEMENTARY TEXT

Device fabrication details

The borosilicate glass substrate was first cleaned using Piranha solution (3:1 ratio of sulfuric acid and hydrogen peroxide), followed by electron-beam evaporation (PVD 75 Electron Beam Evaporation Tool, Kurt J. Lesker Company®, PA, USA) to create a Ti and Au layer. The thicknesses of Ti and Au are 20 nm and 100 nm, respectively. Photolithography using a photoresist (AZ-5214, MicroChem®, Westborough, MA, USA) was then conducted by spin-coating the photoresist (1st spinning: 500 rpm, 10 sec; 2nd spinning: 4,000 rpm, 30 sec), then prebaking the substrate on a hot plate for 1 min (65 °C) and then for 3 min (95 °C). Lithography was conducted using an EVG 610 Double-sided Mask Aligner (separation: 10 µm, mode: hard contact, power: 135 mJ), developed by AZ726 MIF developer, and then the Ti layer etched using hydrofluoric acid (HF) and the Au layer etched using Au etchant (Gold Etchant, standard, Sigma-Aldrich, Saint Louis, MO, USA), followed by rinsing with acetone to remove the photoresist[1, 2].

Next, the protective Si3N4 was deposited. Plasma enhanced chemical vapor deposition (PECVD) was used to deposit a layer of Si3N4 (Oxford Plasmalab80Plus, 300 W, 13.56 MHz RF generator, total gas flow (SiH4, NH3, and N2): 150 - 3,000 sccm, pressure: 200 - 2,000 mTorr) on top of the metal layer.

The microfluidic device was fabricated in polydimethylsiloxane (PDMS, Sylgard 184 Dow Corning, MI, USA) using a conventional soft lithography process. To fabricate the master mold, a photoresist (SU-8® 2050, MicroChem, Westborough, MA, USA) was spin-coated (10 sec at 500 rpm, 30 sec at 4,000 rpm), soft baked on a hot plate (95°C, 10 min), patterned using an EVG 610 Double-sided Mask Aligner (separation: 10 µm, mode: soft contact, power: 220 mJ), post-exposure baked (60°C, 2 min, then 95°C, 10 min), and developed by MIF-319. Then, polydimethyl siloxane precursor (PDMS, Sylgard 184, Dow Corning, MI, USA) was mixed with curing agent at a 10:1 ratio, degassed, poured on the master mold in a petri dish to form a height of 15 mm and a diameter of 100 mm, baked in an oven (70°C) for 24 h, and peeled off from the petri dish. The baked PDMS block was punched to form microfluidic diameter of 0.635 mm inlets and outlet. Plasma treatment using an oxygen plasma (Harrick Plasma PDC-001-HP, 18 W for 120 s) was conducted to bond the glass layer with metal to the PDMS microfluidic layer.

Calculation of droplet size using a Matlab® script

In order to calculate the size of droplets, droplet imaging is required. The provided functions, "regionprops", "cat", "imfindcircles", and "viscircles", were employed to find and measure the droplet size. Once droplet sizes were obtained, then the probability density function (PDF) of the size distribution was plotted through an inherited function, "pdf".

1. SUPPLEMENTARY FIGURES

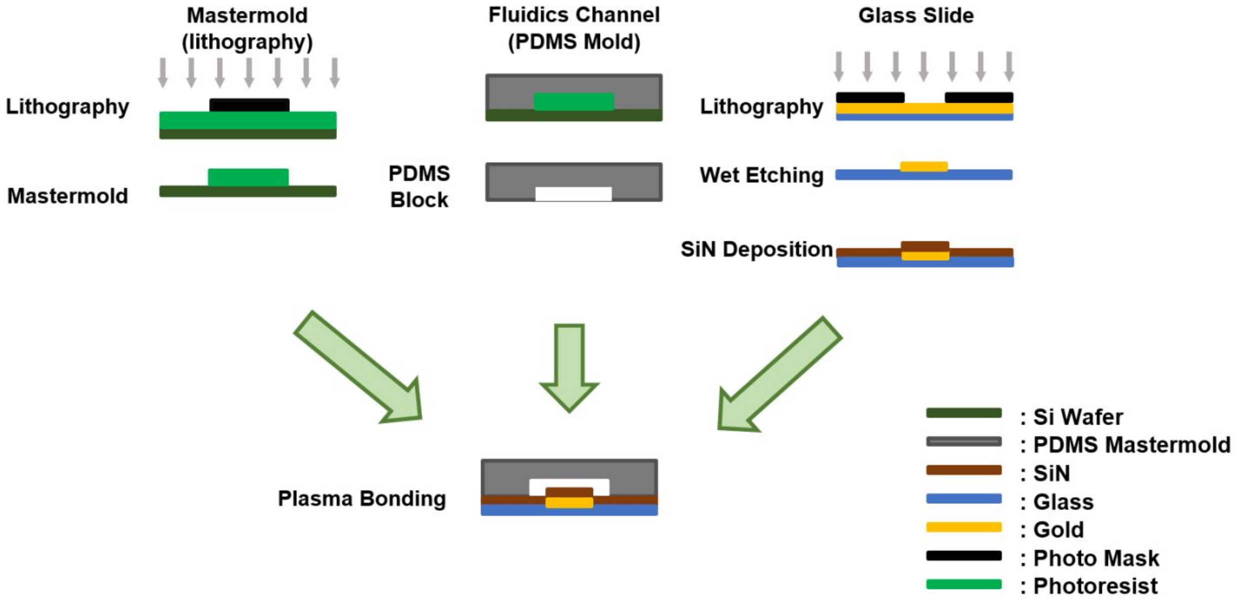

ESI Figure S1. Illustration of the microfabrication steps.

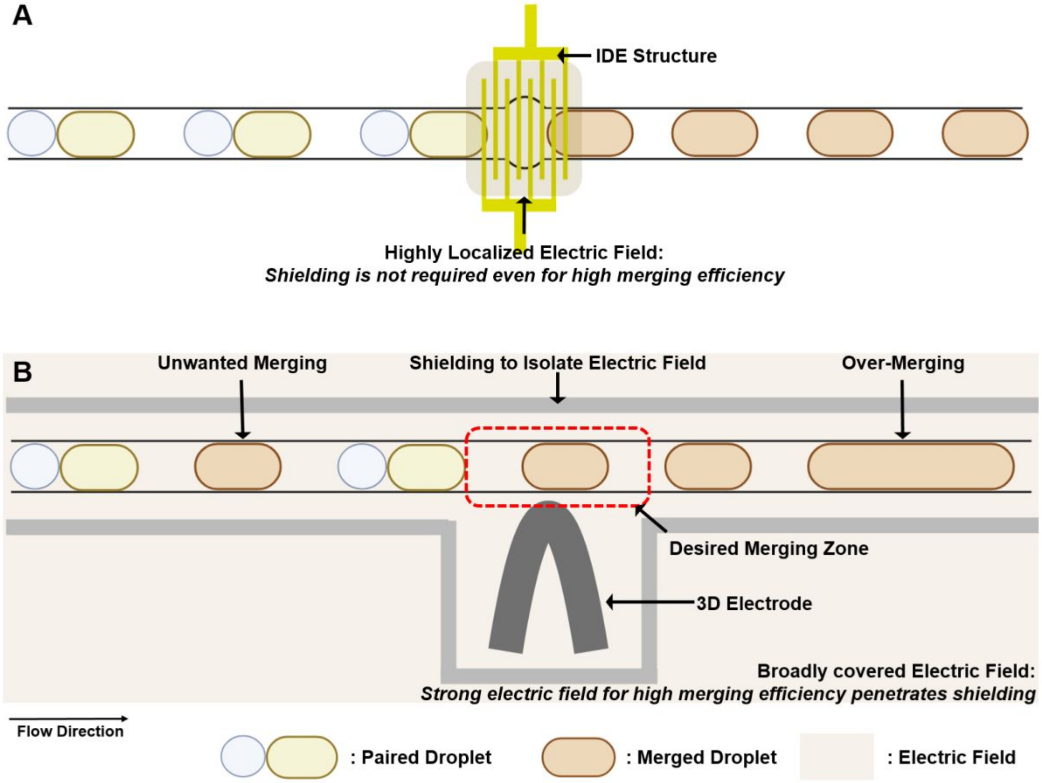

ESI Figure S2. Basic working principle, advantages, and disadvantages of (A) IDE-based droplet merger and (B) conventional droplet merger. A: The IDE droplet merger generates a highly localized electric field to merge paired droplets, eliminating the need for 3D electrode shielding to isolate the electric field, even during high-throughput droplet merging. **B:** A conventional droplet merger creates a strong and extensive electric field to merge paired droplets, often leading to unwanted merging outside the desired merging zone and over-merging.

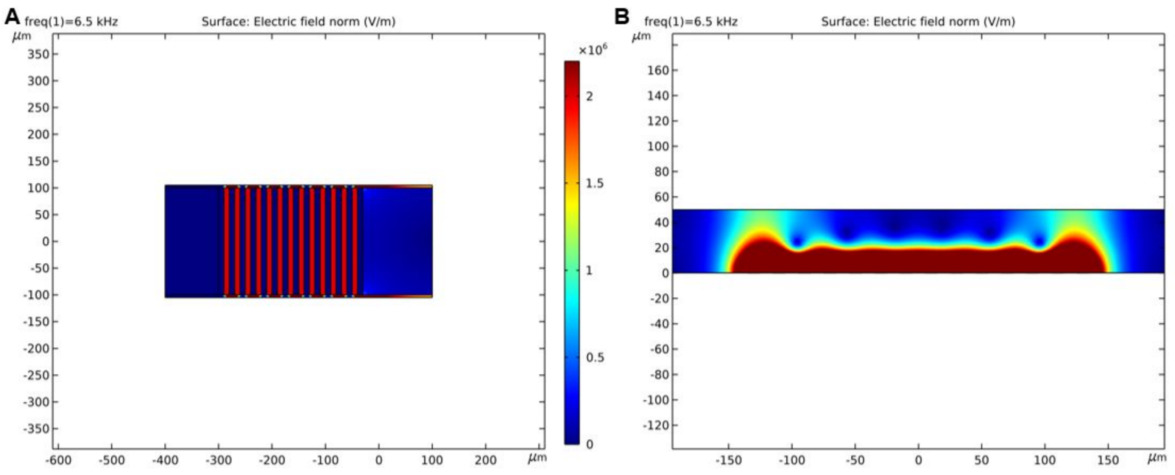

ESI Figure S3. A COMSOL Multiphysics simulation result showing electric field generated by the IDE structure. A: Top view of the electric field showing highly localized electric field that does not affect the droplets outside of the IDE regions. **B:** Side view of the electric field showing that the electric field reaches up to 20-30 µm from the IDE patterns, can physically intact droplets flowing on the IDE structure to merge them.

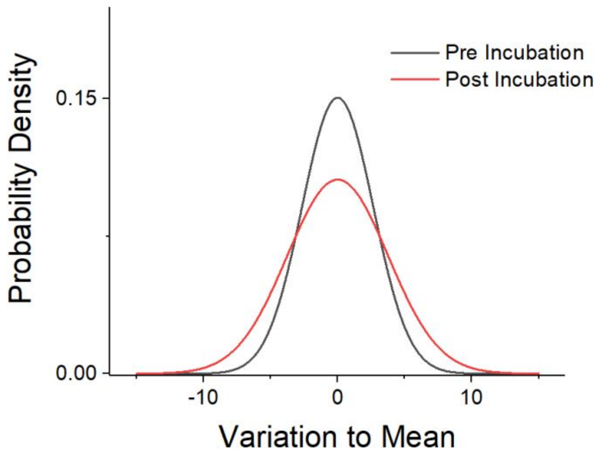

ESI Figure S4. Probability density function (PDF) of the resulting droplet sizes before and after the 12 h incubation step. Average sizes of droplets before and after incubation were 71.6 ± 7.2 µm and 73.2 ± 14.2 µm, respectively.

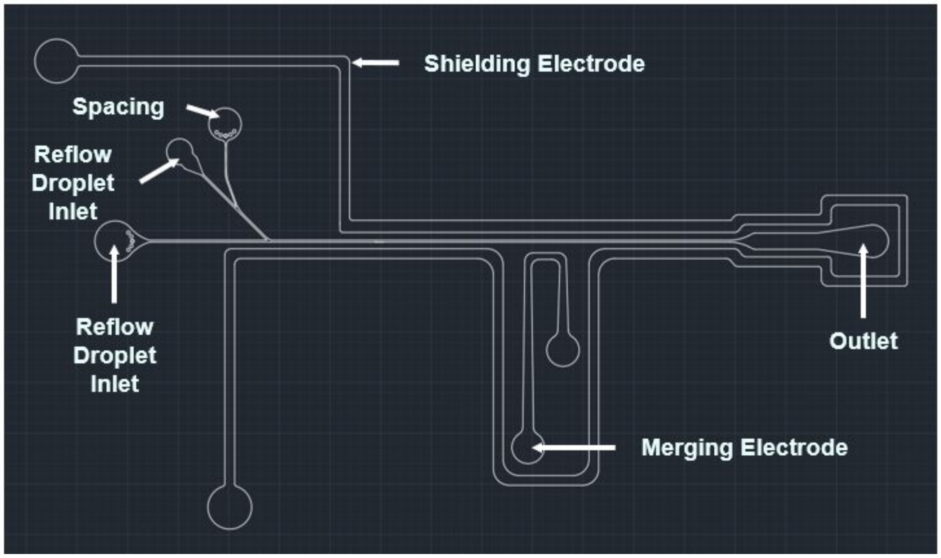

ESI Figure S5. Design of the conventional 3D liquid metal electrode-based droplet merging device used for comparison to the IDE-based droplet merging device.

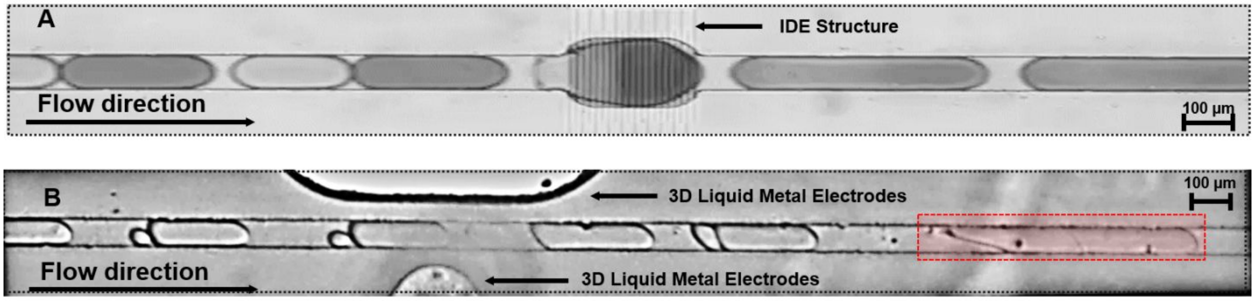

ESI Figure S6. Merging of paired droplets under small droplet-to-droplet spacing using the IDE merger. A: The IDE droplet merging device performs under extremely small droplet pair-to-pair distance (15 µm shown in this figure) without affecting the neighboring droplet pairs due to its highly localized electric field for merging droplets. **B:** The conventional droplet merger device having an 3D electrode requires a larger droplet pair-to-pair distance to avoid over-merging due to its strong and wide electric field. Images within the red box shows an example of an unwanted over-merged droplet outside of the 3D metal electrode. Scale bar = 100 µm.

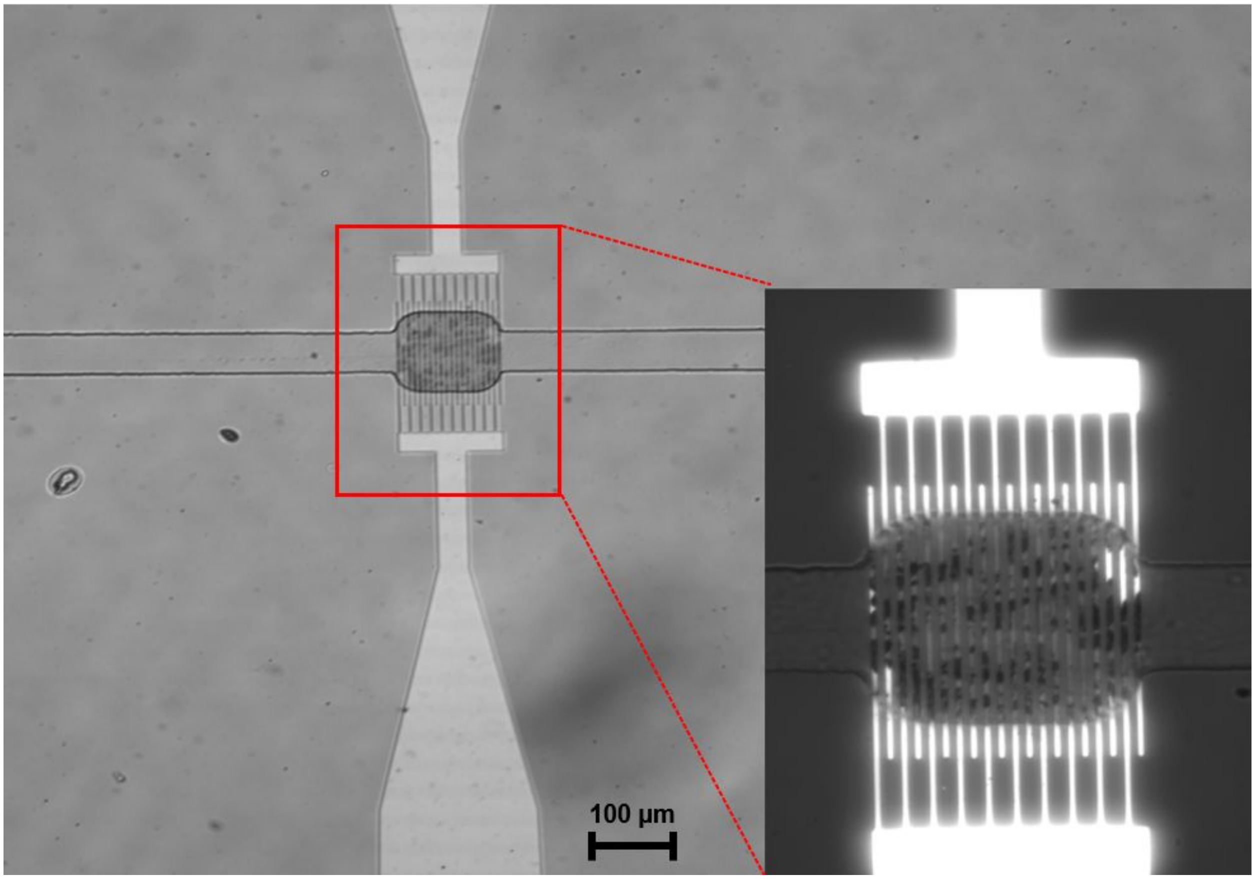

ESI Figure S7. Image of a damaged IDE without Si_3N_4 coating after 4 h of continuous droplet merging operation. Scale bar = 100 µm.

2. SUPPLEMENTARY MOVIES

ESI Movie S1. The expansion channel on the IDE pattern to increase a collision force improving merging efficiency

ESI Movie S2. An example of droplet merging of the IDE droplet merger where a gap between a paired droplets exists.

ESI Movie S3. An example of unwanted merging at the pairing channel of a conventional droplet merger.

ESI Movie S4. Operation of the synchronizing channel of the IDE droplet merger.

ESI Movie S5. Operation of the merging channel of the IDE droplet merger.

ESI Movie S6. The overall operation of the IDE droplet merger.

3. REFERENCE

(1) Li, Y.; Huang, C.; Han, S. I.; Han, A. Measurement of dielectric properties of cells at single-cell resolution using electrorotation. *Biomed Microdevices* **2022**, *24* (2), 23. DOI: 10.1007/s10544-022-00621-3 From NLM Medline.
(2) Han, S. I.; Huang, C.; Han, A. In-droplet cell separation based on bipolar dielectrophoretic response to facilitate cellular droplet assays. *Lab Chip* **2020**, *20* (20), 3832-3841. DOI: 10.1039/d0lc00710b From NLM Medline.
(3) Olsen, N. S.; Hendriksen, N. B.; Hansen, L. H.; Kot, W. A New High-Throughput Screening Method for Phages: Enabling Crude Isolation and Fast Identification of Diverse Phages with Therapeutic Potential. *Phage (New Rochelle)* **2020**, *1* (3), 137-148. DOI: 10.1089/phage.2020.0016 From NLM PubMed-not-MEDLINE.
(4) Hadas, H.; Einav, M.; Fishov, I.; Zaritsky, A. Bacteriophage T4 development depends on the physiology of its host Escherichia coli. *Microbiology (Reading)* **1997**, *143 (Pt 1)*, 179-185. DOI: 10.1099/00221287-143-1-179 From NLM Medline.
(5) Bryan, D.; El-Shibiny, A.; Hobbs, Z.; Porter, J.; Kutter, E. M. Bacteriophage T4 Infection of Stationary Phase E. coli: Life after Log from a Phage Perspective. *Front Microbiol* **2016**, *7*, 1391. DOI: 10.3389/fmicb.2016.01391 From NLM PubMed-not-MEDLINE.
(6) Sciambi, A.; Abate, A. R. Accurate microfluidic sorting of droplets at 30 kHz. *Lab Chip* **2015**, *15* (1), 47-51. DOI: 10.1039/c4lc01194e From NLM Medline.
(7) Li, Y.; Dai, J.; Shim, W.; Han, A. Microfluidic droplet-based high-throughput screening of filamentous fungi. *2022 IEEE Sensors* **2022**. DOI: 10.1109/SENSORS52175.2022.9967267.
